# Clarification of the Cut-off Score for Nine-Item Internet Gaming Disorder Scale–Short Form (IGDS9-SF) in a Chinese Context

**DOI:** 10.3389/fpsyt.2020.00470

**Published:** 2020-05-25

**Authors:** Lixia Qin, Limei Cheng, Maorong Hu, Qiaosheng Liu, Jianqin Tong, Wei Hao, Tao Luo, Yanhui Liao

**Affiliations:** ^1^Department of Psychology, Hospital of Tsinghua University, Beijing, China; ^2^Department of Psychology, Yingtan People's Hospital, Yingtan, China; ^3^Department of Psychiatry, the First Affiliated Hospital of Nanchang University, Nanchang, China; ^4^Department of Psychology, Jiangxi Mental Hospital of Nangchang University, Nanchang, China; ^5^Department of Psychiatry, the Second Xiangya Hospital, Central South University, Changsha, China; ^6^Department of Psychiatry, Sir Run Run Shaw Hospital, School of Medicine, Zhejiang University, Hangzhou, China; ^7^Key Laboratory of Medical Neurobiology of Zhejiang Province, Hangzhou, China

**Keywords:** Internet gaming disorder, Internet Gaming Disorder Scale–Short-Form, cutoff score, clinical settings, universities

## Abstract

**Background:**

The nine-item Internet Gaming Disorder Scale–Short-Form (IGDS9-SF) is a self-reported screening measure based on the Diagnostic and Statistical Manual of Mental Disorders (DSM-5) criteria. It has been used to assesses symptoms and prevalence of Internet Gaming Disorder (IGD) in general population. Despite its widespread use, there is confusion arising from the recommended cutoff score for a positive diagnosis. This study aimed to identify the appropriate cutoff score for IGDS9-SF in a Chinese context.

**Methods:**

The present study included a sample from clinical settings (n = 131) and another from universities (n = 3742). IGDS9-SF measurement and structured clinical interviews based on DSM-5 criteria for IGD were conducted in the sample from clinical settings. The cutoff score was determined using the receiver operating characteristics (ROC) curve. The validity of this cutoff score was further assessed in a sample from universities.

**Results:**

Mathematical models suggest that the score of 32 is the optimal cutoff point (Youden's index, 96.2%; diagnostic accuracy, 96.1%; sensitivity, 98.0%; speciﬁcity, 91.9%; NPV, 91.9%; and NPY, 100%). The prevalence of IGD is 2.9% in this study.

**Conclusion:**

This study suggested that the optimal cutoff score of IGDS9-SF is 32 for the positive diagnosis of IGD in a Chinese context.

## Introduction

Internet gaming disorder (IGD) has been described as a preoccupation with online/offline gaming characterized by impaired control over gaming behaviors that take precedence over other life interests and daily activities despite increasing negative consequences to the individual's psychosocial functioning for a period of at least 12 months (1). According to the Diagnostic and Statistical Manual of Mental Disorders-5 (DSM-5), the clinical diagnosis of IGD requires the endorsement of at least five (or more) of the following nine criteria: 1) preoccupation with online/offline gaming; 2) experience of unpleasant symptoms when gaming is taken away; 3) the need to spend increasing amounts of time engaged in games; 4) unsuccessful attempts to control participation in games; 5) loss of interest in previous hobbies and entertainment as a result of, and with the exception of, games; 6) continued excessive use of games despite knowledge of psychosocial problems; 7) deceiving family members, therapists, or others regarding the amount of gaming; 8) use of games to escape or relieve negative moods; and 9) jeopardizing or losing a significant relationship, job, or education or career opportunity because of participation in games (1). In addition to its negative influences of their social functions, individuals with IGD also displayed the alternation of brain function (2, 3). Early diagnosis of IGD may increase the chances for successful treatment.

Diagnosing IGD in accordance with the DSM-5 requires a clinical interview, but this process is both expensive and time-consuming. As a result, many researchers have developed multiple psychometric questionnaires to screen IGD for clinical and nonclinical settings. The nine-item Internet Gaming Disorder Scale–Short-Form (IGDS9-SF) developed by Pontes and Griffiths (4) is a short psychometric tool based on the nine core criteria defining IGD as suggested by the DSM-5. The IGDS9-SF assesses symptoms and prevalence of IGD by examining both online and/or offline gaming activities occurring over a 12-month period. The scale has been widely used, particularly in the research context. The scale produces final scores between 9 and 45. However, questions have been raised about the appropriateness of cutoff scores, especially when it has been used in different language context.

Internet use and video-game playing are increasing available and accessible for both youth and adult populations in China. A minority of users may experience Internet or Internet gaming addiction. The rate of Internet addiction was higher among Chinese than their counterparts in other countries, like the United States (5). So far, there is, however, no research on the cutoff score of IGDS9-SF in a Chinese context, as well as the prevalence of IGD in adolescents and adults in China. This study aims to examine the optimal cutoff score for the IGDS9-SF in a Chinese context.

## Methods

### Participants and Procedure

The study included samples from clinical settings (n = 131) and from universities (n = 3,742). Clinical samples of were collected from the outpatient clinics of three hospitals (the Department of Psychology of Tsinghua University Hospital, Beijing, China; the Department of Psychology of Yingtan people's Hospital, Jiangxi, China; and the Department of Psychology of Jiangxi Mental Hospital of Nanchang University, Jiangxi, China) between November 12, 2019, and January 7, 2020. The sample from outpatient clinics was composed of 131 participants who were 15- to 35-year-old (M = 18.70, SD = 3.29; male = 74.8%) with problematic gaming behaviors as their chief complaint. After the psychometric assessment of IGD, a blind structured interview was conducted individually by two certified psychiatrists (LQ and TL) to validate and determine the cutoff score of IGDS9-SF.

The sample size assessment for samples from clinical settings are mainly based on the primary outcome of a sample of 555 Brazilian gamers using IGDS9-SF (6). A subsample of 18.9% (n = 104) participants were selected to conduct a clinical evaluation by the certified psychiatrists. Among them, 31.7% was classified as positive diagnosis (4.8% participants diagnosed with IGD and 26.9% at risk) and 68.3% as negative diagnosis (not endorsing at least five out of the nine IGD criteria). Thus, this study aimed to recruit a sample no less than 104 participants. We finally recruited a sample of 131 participants from the outpatient clinics.

To confirm the validity of the IGDS9-SF cutoff score proposed in this study, data collection was also conducted in the classroom setting at three public universities in Beijing, China, during the same period. Students were selected based on availability (convenience sampling). The sample from universities was composed of 3724 participants who were 17- to 35-year-old (M = 20.31, SD = 2.87; male = 44%).

### Ethics

Informed consent was obtained from all participants, while parents' permission was also obtained for those less than 18 years of age. The procedures were carried out in accordance with the Declaration of Helsinki. The ethical approval for this study was also obtained from the Medical Ethics Committee of Tsinghua University.

### Measures

In addition to the collection of demographic information (i.e., gender, age) and relevant characteristics of IGD (i.e., weekly online gameplay time), the survey involved completion of the following scales.

#### Chinese Version of the IGDS9-SF

The Chinese version of the IGDS-9SF Test was self-administered by the participants to assess their IGD symptom severity (7, 8). The test has a 5-point Likert response scale (ranging from 1 = never to 5 = very often). The final score ranging from 9 to 45, with higher scores being indicative of a higher degree of disordered gaming. In this study, the IGDS9-SF presented with a high internal consistency (Cronbach α was 0.91).

#### DSM-5 Diagnostic Criteria of IGD

The diagnostic features of the IGD in DSM-5 comprise nine criteria reflecting its key aspects, including: preoccupation, withdrawal, tolerance, loss of control, give up other activities, continue despite problems, deception, escape, and negative consequences. A structured interview schedule was developed to examine the DSM-5 criteria of IGD based on two recent international recommendations (9, 10). The diagnosis for IGD in the structured interview was based on the cutoff value of 5 suggested in the DSM-5.

### Statistical Analysis

The diagnostic ability of the IGDS9-SF for IGD was evaluated through a ROC analysis using SPSS 25.0 for Windows. The area under the ROC curve (AUC) is a measure of the diagnostic efficacy of IGDS9-SF. The sensitivity, specificity, and Youden's index of IGDS9-SF were evaluated for the diagnostic positive and negative groups. In order to explore the probability that the IGDS9-SF would give the correct “diagnosis,” we also calculated the positive predictive values (PPVs), the negative predictive values (NPVs), and the accuracy values for each possible IGDS9-SF cutoff scores. PPV was defined as the proportion of individuals with positive test results who are correctly diagnosed (11, 12). The NPV was defined as the proportion of individuals with negative test results who are correctly diagnosed (11, 12). The cutoff score of IGDS9-SF is optimal for diagnosis when the score is accompanied by the highest diagnostic accuracy and the optimal Youden's index.

To confirm the validity of the IGDS9-SF cutoff score proposed in this study, school sample were further classified into IGD and non-IGD groups according to the cutoff score of the IGDS9-SF. The demographic information and relevant characteristics of IGD were further compared between these two groups using a χ^2^ test or t-test. A *p* value < 0.05 was considered statistically significant. The analyses were conducted using SPSS 25.0 for Windows.

## Results

Sample characteristics, including gender, levels of education, occupation, marital status, age, IGDS9-SF score, and weekly gaming (hours), were shown in **Table 1**.

**Table 1:** Sample characteristics.

	Clinical sample (n = 131), n (%)	University students sample (n = 3,724), n (%)
Gender		
Male	98 (74.80)	1,638 (22.72)
Female	33 (25.20)	2,086 (51.63)
Education level		
Primary school and below	0	–
Middle school	109 (83.20)	–
High school and above	22 (16.80)	–
Occupational status		
Full time	22 (16.80)	–
Part time	10 (7.60)	–
Unemployed	99 (75.60)	–
Marital status		
Unmarried	127 (96.90)	3,605 (96.80)
Married	3 (2.30)	119 (3.20)
Divorced	1 (0.80)	0
	M (SD)	M (SD)
Age	18.70 (3.29)	20.31 (2.87)
IGDS9-SF score	29.51 (8.54)	14.67 (6.80)
Weekly gaming (hours)	26.00 (14.07)	8.14 (10.80)

### Determination of Cutoff Score

**Figure 1:**
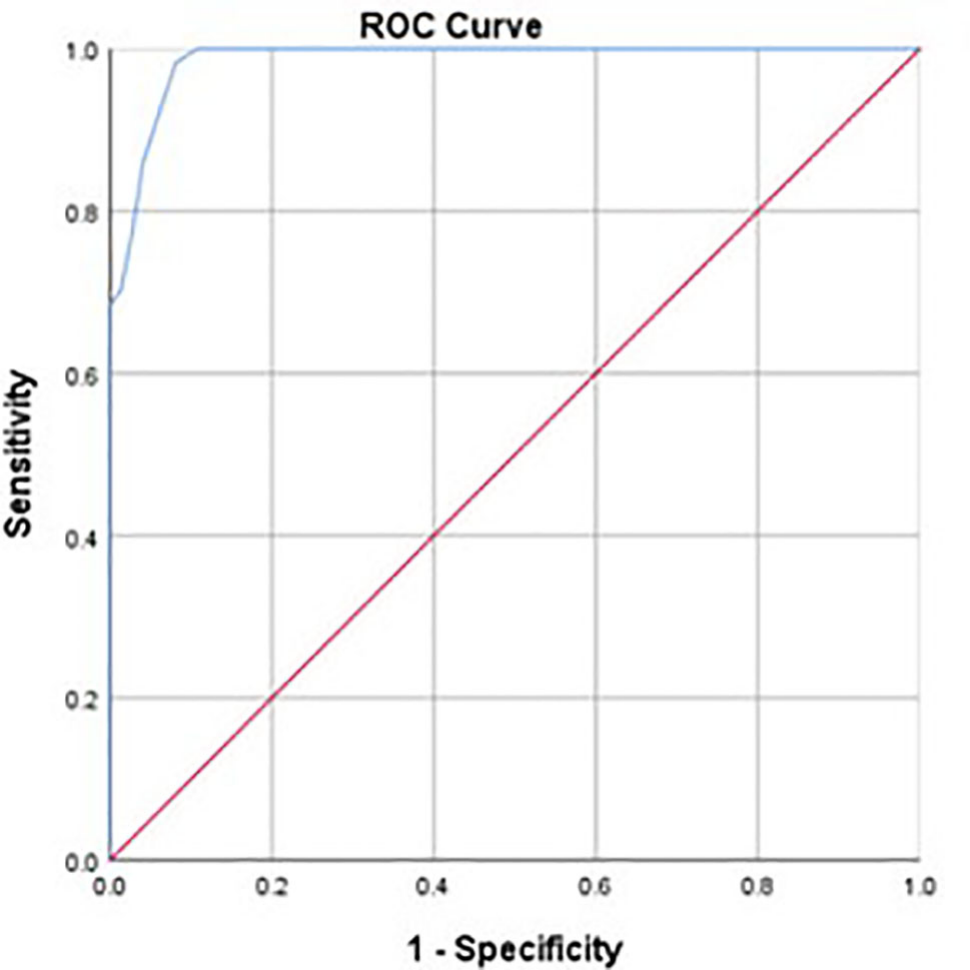
ROC curve (blue line) obtained from clinical sample (AUC, 0.986). ROC, receiver operating characteristic; AUC, area under the ROC curve.

To determine the cutoff score of IGDS9-SF, structured interviews based on the DSM-5 diagnostic criteria for IGD were used as the gold standard. Fifty-seven of the 131 participants met the DSM-5 criteria for IGD. **Figure 1** presents the ROC curve that from the samples. The AUC was 0.998 (P < 0.001), evidencing the high diagnostic efficiency of IGDS9-SF.

The sensitivity, specificity, diagnostic accuracy, positive predictive value (PPV), and negative predictive value (NPV) of the IGDS9-SF at possible cutoff scores were calculated, as they are shown in **Table 2**. The maximum value of the Youden Index of 96.2% is achieved by setting the cutoff score for a positive IGD diagnosis at a score of 32 with sensitivity of 98.0% and specificity of 91.9%. Which means, only 2% of the non-disordered cases would be considered as disordered in the clinical setting, while less than 10% of the truly disordered gamers are not identified by this measurement. Additionally, the PPV is 91.9% and NPV is 100% at this cutoff score of 32. In other words, only 8.1% of the individuals with a positive test result would be mistakenly identified, while all individuals with negative test results would be identified correctly. The diagnostic accuracy was 96.1%. Increasing or decreasing the cutoff scores would result in more false negative or false positive cases, and correspondingly decrease the diagnostic accuracy.

**Table 2:** Cutoff points in IGDS9-SF between diagnostic positive and negative groups (N = 131).

Cutoff	True positive	True negative	False positive	False negative	Sensitivity (100%)	Specificity (100%)	PPV (100%)	NPV (100%)	Accuracy (100%)	Youden's Index (100%)
21	57	37	37	0	100	50.0	60.6	100	71.7	50
29	57	58	15	0	100	78.4	78.0	100	87.7	78.4
30	57	65	8	0	100	87.8	86.3	100	93.1	87.8
31	57	69	7	0	100	89.2	87.6	100	93.8	89.2
**32**	**57**	**74**	**5**	**0**	**98.0**	**91.9**	**91.9**	**100**	**96.1**	**90.1**
33	52	74	0	5	86.0	95.9	100	93.6	96.1	81.9
34	46	74	0	11	77.2	97.3	100	87.0	91.6	74.5
35	41	74	0	16	70.2	98.6	100	82.2	87.1	68.8
36	39	74	0	18	68.0	100	100	80.4	86.2	68.0
A^*^	42	74	0	15	73.6	100	100	83.1	88.5	73.6

IGDS9-SF, Nine-Item Internet Gaming Disorder Scale–Short-Form; sensitivity, the proportion of true positive rates; speciﬁcity, the proportion of the true negative rates; PPR, positive predictive rate; NPR, negative predictive rate; Youden's Index, deﬁned as sensitivity + speciﬁcity − 1; AUC = 0.998.

A*: indicated the cutoff point of five or more of the nine IGD criteria on the basis of answering “very often.”

At the cutoff score of 36 recommended by Pontes et al. (4), the sensitivity is 68.0%, the specificity is 100%; or at the point of five or more of the nine IGD criteria on the basis of answering “Very often” (4), the sensitivity is 73.6%, the specificity is 100%. Comparatively, the sensitivity of the cutoff of 21 recommended by Monacis et al. (13) and Severo et al. (6) is 100%, while the specificity of 50.0%.

### IGD Prevalence Estimate

Based on the cutoff score of 32 for classifying disordered gamers, IGD prevalence estimate in the present sample was 2.9% (95% CI = 2.4–3.4). The prevalence of IGD was 4.5% (95% CI = 3.5–5.5) in male students, which was higher than in female students (1.7%, 95% CI = 1.1–2.2, χ^2^ = 25.16, *p* < 0.001). **Table 3** presents the group comparisons of demographic data and relevant characteristics of IGD. Significant gender differences have been identified between the IGD and non-IGD groups. Compared with the non-IGD group, the IGD group exhibited significantly more weekly gaming time.

**Table 3:** Comparison between the IGD and non-IGD groups according to cutoff point of 32 in the IGDS9-SF (N = 3,724).

	IGD (*N*=108)		Non-IGD (*N*=3616)		*χ^2^*
	*N*	%	*N*	%	
Gender					25.16^**^
Male	73	67.59	1565	43.28	
Female	35	32.41	2051	56.72	
	*M*	*SD*	*M*	*SD*	*t*
Age	20.44	3.34	20.31	2.85	0.49
Weekly game time	27.99	8.90	7.54	5.28	20.44^**^

IGD, Internet gaming disorder; IGDS9-SF, nine-item internet gaming disorder scale–short-form.

**p < 0.001.

## Discussion

The study revealed a clinically optimal cutoff score of 32 for diagnosing IGD with the IGDS9-SF test in the Chinese context. This point represents the best balance between sensitivity (98.0%) and specificity (91.9%), and the highest diagnostic accuracy (96.1%). The cutoff point 32 in this study is lower than that in Pontes' study (with a sample of 1397 English-speaking gamers from 58 different countries) (4), which recommended a cutoff point of 36 or over, or five or more of the nine IGD criteria on the basis of answering “very often.” In Pontes' study, the sensitivity of this two cutoff points is relatively low (68.0% and 73.6% respectively), although the specificity is 100%, which is the same as our study. The results from Pontes' study indicate that approximately one in three or four disordered cases would not be identified. This may be considered unacceptable in an epidemiological screening context.

Monacis et al. (13) suggested that a score of 21 might be more appropriate for use in an Italian context. This point was confirmed by the study of Severo et al. (6) in a Brazilian context. The Italian sample recruited a sample of 687 participants, including 375 males and 312 females (mean age  =  21.62 years, SD  =  3.90), from schools, universities, and gaming halls. The receiver operating characteristics (ROC) analysis resulted in a cutoff point of 21 in determining IGD, and the AUC (area under the curve) of the Italian version of the IGDS9-SF was 0.935 (13). The Brazilian sample recruited a total of 555 participants, including 319 males and 236 females (mean age = 20.3 years, SD = 5.4), from a location where the individuals are highly exposed to computers and technology. ROC plot analysis also yielded a cutoff point of 21, with an AUC of 0.935 for IGD positive (6).

However, at the cutoff score of 21 suggested by Monacis et al. (13) and Severo et al. (6), the specificity is only 50%, meaning one half non-disordered cases would be considered disordered. The cutoff score of 21 from these two studies would be quite inappropriate if applied to the Chinese context, as it may lead to overestimation of the prevalence of IGD.

The prevalence estimate rate in this study is 2.9%, with relatively high consistency with earlier studies in Chinese context (14, 15). Our results also reveal a significant gender difference in IGD. Male students were more likely to meet the IGD criteria, which consistent with other culture studies (16–19). Furthermore, probable disordered gamers showed more weekly gaming time as expected. These results indicate that the cutoff score of 32 appropriately differentiated Chinese gamers.

These findings should be considered in the light of the limitations, that all participants were recruited on the basis of their availability (i.e., convenience sample). Consequently, the findings of prevalence rate need to be cautiously interpreted in terms of generalizability. Also, this study mainly recruited young adults (age ≤ 35 years old), and only a very small number of adolescents. Thus, we did not examine the differences of the prevalence of IGD between adolescents and young adults. Future studies should aim to reveal the prevalence of IGD in representative samples across different age groups. Furthermore, the sole use of self-report questionnaires in the sample from universities has some inherent disadvantages, such as memory bias.

## Conclusions

In sum, the potential value of the IGDS9-SF as a screener for IGD is evidenced by the above results, including the high value registered for the area under the ROC curve and sensitivity and specificity figures. Based on our findings, we recommend the use of an IGDS9-SF score of 32 as the cutoff point for the positive diagnosis of IGD in a Chinese context.

## Data Availability Statement

The datasets generated for this study are available on request to the corresponding authors.

## Ethics Statement

Informed consent was obtained from all participants, while parents' permission was also obtained for those less than 18 years of age. The procedures were carried out in accordance with the Declaration of Helsinki. The ethical approval for this study was also obtained from the Medical Ethics Committee of Tsinghua University.

## Author Contributions

TL and LQ conceived the study. TL, MH, QL, JT, and YL did the literature review, statistical analyses, and drafted the report. TL and LQ collected the data. All the authors interpreted the data and commented on the manuscript. All the authors contributed to and have approved the final manuscript.

## Funding

This study was supported by the Natural Science Foundation of Jiangxi Province of China (No. 20192BAB205037), and the “Hundred Talents Program” funding from Zhejiang University.

## Conflict of Interest

The authors declare that the research was conducted in the absence of any commercial or financial relationships that could be construed as a potential conflict of interest.
